# Subcutaneous Separation of Trachea and Larynx

**Published:** 1888-04

**Authors:** 


Subcutaneous Separation of Trachea and Larynx.—Noll, of Hanau, reported at the sixtieth Versammlung Deutscher Naturforscher und Aerzte, in September, 1887, the case of a workman who received a blow from a piece of machinery on the front part of his neck. The skin was not wounded, and only a little blood was coughed up. The neck soon became very much swollen, and attacks of suffocation came on. On performing tracheotomy Noll found that the trachea was separated from the larynx, and was very much retracted, and that the cricoid and thyroid cartilages were fractured. The trachea was drawn up and sutured to the larynx, and a cannula introduced. Later, this could not be removed because of cicatricial contraction. The usual methods of dilatation failed, and a laryngo-fissure was made, in which a Dupuis' cannula was worn for nine months. The trachea and larynx are now completely grown together, and the patient is in as good condition as before, except that his voice is somewhat hoarse—*Deutsche med. Wochenschrift*, No. 52, 1887.

